# Bayesian joint modelling of longitudinal and time to event data: a methodological review

**DOI:** 10.1186/s12874-020-00976-2

**Published:** 2020-04-26

**Authors:** Maha Alsefri, Maria Sudell, Marta García-Fiñana, Ruwanthi Kolamunnage-Dona

**Affiliations:** 1 Department of Health Data Science, Institute of Population Health, University of Liverpool, L69 3GL, Liverpool, UK; 2 Department of Statistics, University of Jeddah, Jeddah, Saudi Arabia

**Keywords:** Joint models, Longitudinal outcomes, Time-to-event, Dynamic prediction, Bayesian estimation

## Abstract

**Background:**

In clinical research, there is an increasing interest in joint modelling of longitudinal and time-to-event data, since it reduces bias in parameter estimation and increases the efficiency of statistical inference. Inference and prediction from frequentist approaches of joint models have been extensively reviewed, and due to the recent popularity of data-driven Bayesian approaches, a review on current Bayesian estimation of joint model is useful to draw recommendations for future researches.

**Methods:**

We have undertaken a comprehensive review on Bayesian univariate and multivariate joint models. We focused on type of outcomes, model assumptions, association structure, estimation algorithm, dynamic prediction and software implementation.

**Results:**

A total of 89 articles have been identified, consisting of 75 methodological and 14 applied articles. The most common approach to model the longitudinal and time-to-event outcomes jointly included linear mixed effect models with proportional hazards. A random effect association structure was generally used for linking the two sub-models. Markov Chain Monte Carlo (MCMC) algorithms were commonly used (93% articles) to estimate the model parameters. Only six articles were primarily focused on dynamic predictions for longitudinal or event-time outcomes.

**Conclusion:**

Methodologies for a wide variety of data types have been proposed; however the research is limited if the association between the two outcomes changes over time, and there is also lack of methods to determine the association structure in the absence of clinical background knowledge. Joint modelling has been proved to be beneficial in producing more accurate dynamic prediction; however, there is a lack of sufficient tools to validate the prediction.

## Background

Over the last decade, there has been an increasing interest in joint models for longitudinal and time-to-event outcome data, especially in medical research, due to their ability to predict individual-level patients’ risks. A joint model consists of two linked sub-models. The relationship between the longitudinal and time-to-event outcomes is represented by an association structure, a function that links the longitudinal and time-to-event sub-models. A commonly used longitudinal sub-model is the linear mixed effect model, and the time-to-event sub-model is often the Cox proportional hazards model.

Joint modelling reduces the biases of parameter estimates by accounting for the association between the longitudinal and time-to-event data [1]. In clinical trials, this leads to more efficient estimation of the treatment effect on both time-to-event and longitudinal outcomes. It also quantifies the strength of the association between longitudinal and time-to-event outcomes. Joint models have been used in several areas in the medical literature to study the relation between longitudinal biomarkers and a time-to-event of interest, e.g. AIDS studies [2–4] and cancer [5, 6].

Estimating the effect of longitudinal outcomes on the risk of the event can be carried out using a frequentist or Bayesian approach. While frequentist approaches are common and well understood, employing a Bayesian approach to joint models allows for a more flexible estimation as well as using related historical information may improve the analysis. The maximum-likelihood approach is the standard estimation approach in frequentist framework [7], while the Bayesian approaches are generally based on Markov chain Monte Carlo (MCMC) sampling algorithms (e.g. [8, 9]). The methods and inference of the joint model in general are explained in details in several tutorial papers [10–12].

A review of joint models primarily focussing on frequentist approaches was carried out by Hickey et al. [1]. However, due to the recent popularity of data-driven approaches in medical research, there is a need for a comprehensive review of joint models under the Bayesian framework. In this review, we summarise currently available methodology, fitting algorithms, dynamic prediction approaches and software for joint models proposed within the Bayesian framework.

## Methods

**Fig. 1:**
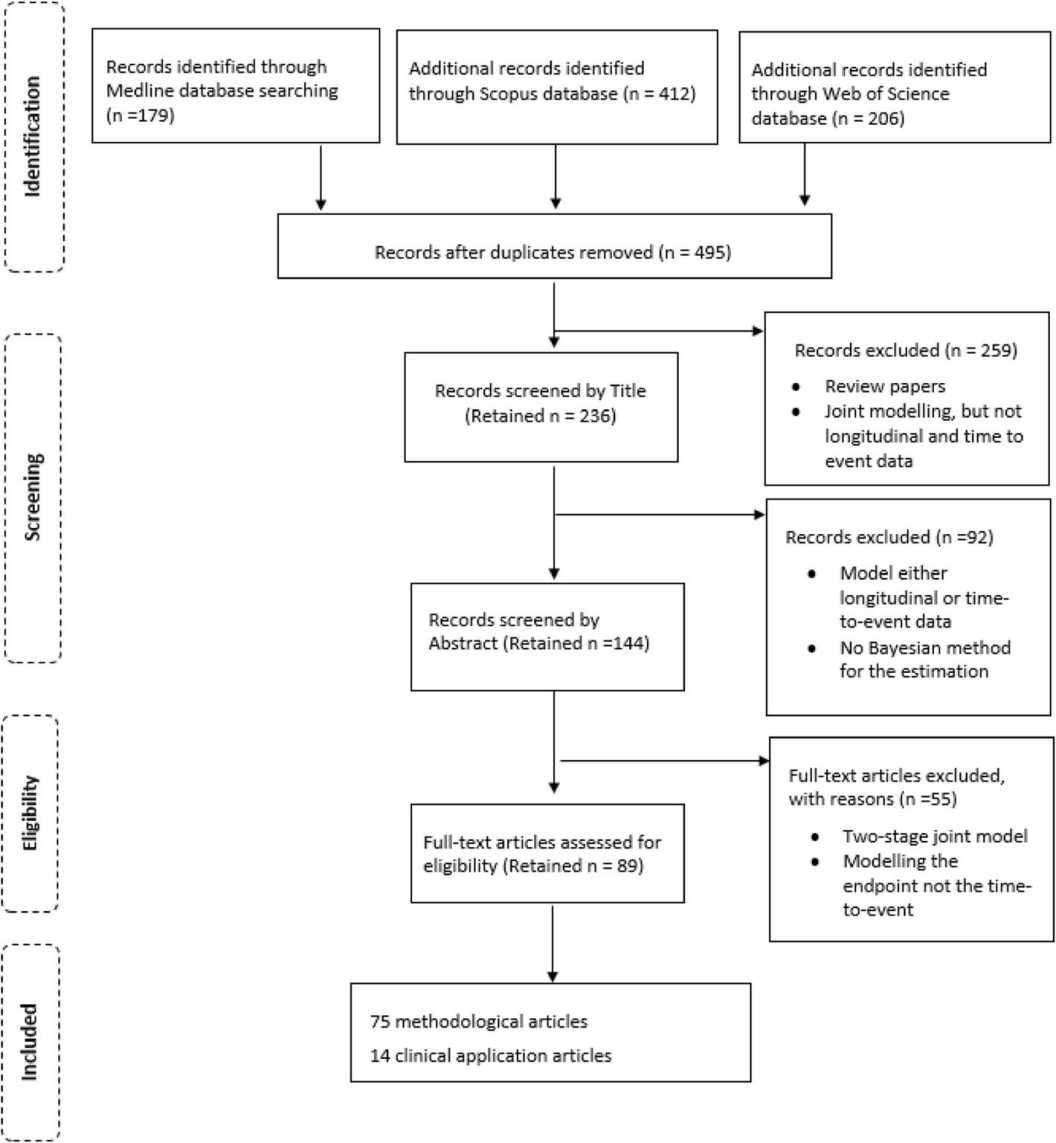
Flowchart of study identification

The search included articles published up to July 2019, and we have searched in three databases; Medline, Scopus and Web of Science. The study identification journey is shown in Fig. 1. In each database, four different keyword searches were applied to identify the articles; “joint model AND Bayesian” or “joint models AND Bayesian” or “joint modelling AND Bayesian” or “ (longitudinal and survival) AND Bayesian”. To identify the target articles, these keywords were searched in the title and abstract in Medline and Scopus databases, and in the title in Web of Science. The complete search strategy is available in the Additional file 1 and a blank data extraction form is presented in Additional file 2.

A total of 797 articles were identified from the search, with 179, 412 and 206 articles resulted from each of Medline, Scopus and Web of Science respectively. Duplicates were identified and removed, leaving 495 articles. The lead author screened all articles, and if an article could not be determined whether to include or not, it went to a voting procedure by the rest of the authors. Based on screening of the article title, 236 were found relevant. The excluded articles included joint models other than longitudinal and event-time (e.g. multiple longitudinal outcomes alone), and review articles. A further 92 articles were excluded as a result of screening the abstract. This exclusion included articles that were only modelling longitudinal data or event-time data, and articles that did not use a Bayesian approach for parameter estimation.

Full-text articles were obtained for the remaining 144 and reviewed in full. A total of 55 articles that used two-stage joint model were excluded (where the longitudinal and event-time outcomes are modelled in two separate steps rather than simultaneously), and if a dropout process is modelled, but not as an event-time outcome. Finally, a total of 89 articles were eligible for inclusion in the review. The articles were sorted into methodological and application groups, containing 75 articles and 14 articles respectively. A methodological article was classed as one that proposed and demonstrated new methodology whereas the application articles were classed as those that applied existing methodology to a new dataset. In the following sections, the identified methodological articles are reviewed.

## Results

We have found the joint modelling methods developed under the four categories: single outcome for both of the longitudinal and event-time data (39/75, 52%); single longitudinal outcome and multiple event-time outcomes (13/75, 17.3%); multiple longitudinal outcomes and single event-time outcome (15/75, 20%); both outcomes are multiple (8/75, 10.7%). The majority of the articles were based on shared random effect joint models [13–66], whereas several articles explored joint models in terms of latent classes [42, 54, 58, 67–70], additive model [71, 72] and functional model [73, 74]. We reviewed the methodology for each sub-model and association structure.

### Longitudinal data sub-model

Let *Y*_*ik*_(*t*_*ijk*_) denote the *jth* observed value of the *k*-th longitudinal outcome for the individual *i* at time *t*_*ijk*_ for *i* = 1, …, *N*; *k* = 1, …, *K* and *j* = 1, …, *n*_*ik*_ , with *N* is the total number of individuals in the study , *n*_*ik*_ is the total number of measurements for the *k* th longitudinal outcome of individual *i* and *K* is the total number of longitudinal responses in the study. The most common approach to model the longitudinal data was the generalized linear mixed model (GLM) and is given by

$$ {g}_k\left(E\left\{{Y}_{ik}\left({t}_{ijk}\right)\right\}\right)={m}_{ik}\left({t}_{ijk}\right) $$

where *g*_*k*_(.) is a known link function of the *k* th longitudinal outcome, *m*_*ik*_(.) is a linear predictor and *E*(.) is the expectation operator. When a single longitudinal outcome is considered, that is when *K* = 1, and for the simplicity of notation, *k* is dropped from the notation.

The majority of articles (48/75, 64%) involved development of joint models with a continuous longitudinal outcome, Table 1 and Table 2. One article proposed a modelling approach for ordinal outcomes [26], and two articles for counts [31, 53]. Modelling for a mixture of outcomes was proposed by He and Luo [30], who modelled continuous, ordinal and binary outcomes in a Parkinson’s disease study. Dagne [44] and Lu [25] proposed an approach to account for longitudinal data with a lower quantification limit (called left censoring in the longitudinal outcome). Of the 75 included articles, 51 (68%) proposed models for a single longitudinal outcome, while the remaining 24 (32%) considered models for multiple longitudinal outcomes (the univariate and multivariate longitudinal models are illustrated in more detail in Additional file 3).

**Table 1:** Summary of longitudinal sub-models with single longitudinal outcome

	Number of articles (%)	Reference
**Type of outcome**		
Continuous	39(95.1%)	[13, 15–19, 22–25, 27, 33, 35, 37–40, 43, 44, 46–48, 51, 52, 55, 56, 59–62, 65, 66, 75–81]
Count	2(4.9%)	[31, 53]
**Model**		
GLM, NLME, SNLME, Semiparametric random-effects model^a^	5(12.2%)	[51, 52, 77, 78, 81]
LME	13(31.7%)	[15–19, 23, 33, 35, 46, 48, 62, 75, 76]
Partially LME	4(9.8%)	[22, 37, 56, 61]
Mixed effect model, Mixed effect model with IOU stochastic process, Mixed-effect varying coefficient Tobit model, Bent-cable mixed-effects model^a^	4(9.8%)	[25, 39, 44, 47]
Mixed-effects varying-coefficient model	3(7.3%)	[27, 38, 80]
LQMM, Quantile-based mixed model, QR- NLME, QR-NLMET^a^	4(9.8%)	[24, 59, 60, 79]
Hurdle two-part model and Longitudinal Tobit model^a^	2(4.8%)	[43, 66]
Random change point model, Multiple-change point model, Longitudinal model for the immune response ^a^	4(9.8%)	[13, 40, 55, 65]
ZAB, Two zero-inflated count models^a^	2(4.8%)	[31, 53]
**Random effect distribution**		
Normal	17(47.4%)	[13, 15, 19, 25, 27, 33, 38, 46, 47, 51, 55, 60, 75–77, 79–81]
Multivariate normal	10(26.3%)	[16–18, 24, 39, 43, 44, 59, 62, 66]
Finite mixture of normal distributions, N/I^a^	3(7.9%)	[48], [23, 35]
Dirichlet process prior	2(5.3%)	[40, 52]
Spline	5(13.1%)	[22, 37, 56, 61, 78]
**Error distribution**		
Normal	18(48.6%)	[13, 16, 17, 19, 33, 39, 40, 46–48, 52, 55, 62, 65, 66, 75, 76, 81]
N/I, SN^a^	3(8.1%)	[23, 35], [51]
t-distribution	1(2.8%)	[18]
ST	6(16.2%)	[15, 22, 37, 38, 56, 61]
Multivariate ST	6(16.2%)	[25, 27, 44, 77, 78, 80]
ALD	3(8.1%)	[24, 59, 79]

^a^The order of the outcomes/models/distributions have the same order as in the reference

**Table 2:** Summary of longitudinal sub-models with multivariate longitudinal outcomes

	Number of articles (%)	Reference
**Type of outcome**		
Continuous	8(36.4%)	[20, 21, 28, 32, 49, 63, 82, 83]
Rate, Ordinal, \ (or/and continuous), Continuous, Ordinal and Discrete^a^	5(22.7%)	[14, 26, 34, 50, 84]
Continues and binary	2(9.1%)	[36, 57]
Continuous and ordinal	3(13.6%)	[29, 41, 45]
Continuous, ordinal and binary	4(18.2%)	[30, 85–87]
**Model**		
GLM, Partially LME^a^	2(9.1%)	[20, 32]
Multivariate GLM	4(18.2%)	[14, 34, 36, 57]
Multivariate mixed effect models	5(22.7%)	[21, 28, 63, 82, 83]
ZAB, Proportional-odds cumulative logit model^a^	2(9.1%)	[26, 50]
GLM and CR mixed-effects model, Mixed-effect model and CR mixed-effects model, LME and continuous latent variable model, LME and a mixed-effects beta regression model, ZOIB^a^	5(22.7%)	[29, 41, 45, 49, 84]
MLIRT	2(9.1%)	[30, 86]
MLLTM, MLTLM^a^	2(9.1%)	[85, 87]
**Random effect distribution**		
Normal	12 (54.5%)	[20, 26, 29, 30, 36, 45, 50, 82, 84–87]
Multivariate normal	7(31.8%)	[14, 21, 28, 41, 49, 63, 83]
Dirichlet process prior	3(13.7%)	[32, 34, 57]
**Error distribution**		
Normal	12(63.2%)	[20, 29, 30, 36, 41, 45, 49, 57, 82, 83, 85, 87]
Multivariate normal SN	4(21.1%)	[14, 21, 34, 63]
Finite mixture of normal distributions, Multivariate SN, SN/I^a^	3(15.7%)	[28, 32, 86]

^a^The order of the outcomes/models/distributions have the same order as in the reference

### Single longitudinal outcome (*K =* 1)

#### Continuous outcome

##### Linear mixed-effect model

Linear Mixed-Effect (LME) models were generally used to model continuous longitudinal data [16, 17, 19, 33, 46, 48, 62, 75, 76, 82], and were defined by

1 $$ {Y}_i\left({t}_{ij}\right)={X}_i\left({t}_{ij}\right)\beta +{Z}_i\left({t}_{ij}\right){b}_i+{\varepsilon}_{ij} $$

where *X*_*i*_(*t*_*ij*_) and *Z*_*i*_(*t*_*ij*_) are covariates (possibly time-varying) matrices for fixed effects *β* and random effects *b*_*i*_ respectively contributing to the linear predictor, and *ε*_*ij*_ is an independent and identically distributed Gaussian measurement (or residual) error.

The assumption of normality for the within individual measurement error can be beneficial in model implementation, however, any outlying observations could influence the statistical inference [23]. Therefore, when this assumption was violated due to skewness or outliers, alternative distributions were proposed, including skew-normal (SN) distribution [15, 51], skew-t (ST) distribution [22, 37, 56, 61], t-distribution [18], or normal/independent (N/I) distribution [23, 35]. N/I distribution is a family of mixture distributions conditional on an independent positive random variable; more details on this type of distribution can be found in Andrews and Mallows [88]. Moreover, the simulation study conducted by Baghfalaki et al. [23] and Baghfalaki et al. [35] has showed the robustness of the chosen N/I distribution against outliers and its unbiasedness as compared with the conventional normal distribution. Huang et al. [77] proposed a nonlinear mixed-effects (NLME) model, and Huang et al. [78] proposed a semiparametric nonlinear mixed-effects (SNLME) model when the longitudinal data follows a ST distribution. Bakar et al. [47] employed an Integrated Ornstein-Uhlenbeck (IOU) stochastic process to model individual variations. This approach is more flexible and plausible than a random effects model as it enables the longitudinal outcome to vary around a straight line and allows the data to determine the degree of this variation.

Brown and Ibrahim [52] proposed a semiparametric linear mixed-effect model. They have used Dirichlet process priors on the parameters defining the longitudinal model. Dirichlet process prior is a process that can be used to create a family of distributions to provide more flexible priors than the standard normal distribution [40]. This approach is proposed when there is uncertainty about the distributional assumptions, and it offers more flexibility in modelling the longitudinal trajectory [52]. For example, individuals in HIV (human immunodeficiency virus) studies and cancer vaccine trials might encounter more diverse longitudinal trajectories due to the variety of treatment response on each individual. In cancer vaccine trials, many patients may not exhibit an immune response to vaccination at varying time points throughout the trial. Therefore, Brown and Ibrahim [65] assumed an initial distribution (called “point mass at zero”) for the baseline immune response, and developed a longitudinal model for the immune response with this point mass at zero, in which the probability that an observation arises from the point mass changes over time and between individuals. The distribution of the response variable is dependent on the response of the patients to vaccination.

##### Quantile modelling

Some clinical studies are interested in making predictions from the joint model on the median or lower/upper ends of the longitudinal trajectory rather than on the mean. In this case, linear quantile mixed model (LQMM) can be used to describe the longitudinal process by

2 $$ {Q}_{Y_i\left({t}_{ij}\right)\mid {X}_i\left({t}_{ij}\right),{Z}_i\left({t}_{ij}\right)}\left(\tau \right)={m}_i^{\tau}\left({t}_{ij}\right)+{\varepsilon}_{ij} $$

where ${m}_i^{\tau }(t)$ is the true underlying value of the longitudinal outcome *Y*_*i*_(*t*_*ij*_) at *τ* th quantile measured at time t,

3 $$ {Q}_{Y_i\left({t}_{ij}\right)}\left(\tau \right)={F}_{Y_i\left({t}_{ij}\right)}^{-1}\left(\tau \right)=\mathit{\operatorname{inf}}\ \left\{{Y}_i\left({t}_{ij}\right):{F}_{Y_i\left({t}_{ij}\right)}\left({Y}_i\left({t}_{ij}\right)\right)\ge \left(\tau \right)\right\}\mathrm{for}\ \uptau\ \upepsilon\ \left[0,1\right] $$

In the above equation, ${F}_{Y_i\left({t}_{ij}\right)}$ is the distribution function of *Y*_*i*_(*t*_*ij*_) and *inf* represents the infimum function. Yang et al. [24] considered a LQMM, whereas Huang and Chen [59] proposed quantile regression based nonlinear mixed-effects model (QR-based NLME) when response trajectories are nonlinear. Waldmann and Taylor-Robinson [60] considered a quantile-based mixed model, an extension to the mean regression joint model proposed by Faucett and Thomas [89]. Yang et al. [24] and Huang and Chen [59] considered independent asymmetric Laplace distribution (ALD) in each time point for the error term in the quantile model since it is robust against outliers or to account for skewness in the longitudinal process. When the longitudinal outcome is measured with limit of detection (see section below) and covariates are skewed with measurement error, Zhang and Huang [79] employed a quantiles regression based nonlinear mixed-effects Tobit (OR-NLMET) model. In this model, the continuous longitudinal outcome assumes an asymmetric Laplace distribution (ALD).

##### Modelling of left-censored longitudinal outcomes

In measuring the longitudinal outcome, some repeated measurements are left-censored due to limit of detection (LOD) [25, 44]. Usually LOD is a threshold defining the minimum value that can be observed, and measurements below the LOD are known as ‘censored’. As the standard LME model does not account for left censoring, Dagne [44] and Lu [25] proposed alternative models to tackle this issue. Lu [25] proposed modelling left-censored longitudinal data using the mixed-effect varying coefficient Tobit model:

4 $$ {\displaystyle \begin{array}{c}{Y}_i\left({t}_{ij}\right)={\beta}_0\left({t}_{ij}\right)+{X}_i^{\ast}\left({t}_{ij}\right){\beta}_{1i}\left({t}_{ij}\right)+{\varepsilon}_{ij}\\ {}{\beta}_{1i}\left({t}_{ij}\right)={\beta}_1\left({t}_{ij}\right)+{b}_i\left({t}_{ij}\right)\end{array}} $$

where *β*_0_(*t*_*ij*_) and *β*_1*i*_(*t*_*ij*_) denote the time-varying coefficients for intercept and slope respectively, and ${X}_i^{\ast}\left({t}_{ij}\right)$ is the true (unobservable) covariate value, and *β*_1_(.) denotes the population smoothing curve while *b*_*i*_(*t*_*ij*_) indicate the random effects.

Dagne [44] considered a bent-cable mixed-effect model to account for two growth curves in the longitudinal data. The model was defined by

5 $$ {Y}_i\left({t}_{ij}\right)=g\left({t}_{ij},{\mu}_{ij},{X}_{ij}^{\ast}\right)+{\varepsilon}_{ij} $$

where *μ*_*ij*_ is the mean structure and ${X}_{ij}^{\ast }$ is the true (unobservable) covariate. To account for between-individual and within-individual variation, Brilleman et al. [43] proposed a hurdle two-part model with first part estimating the probability when the longitudinal outcome is observed above the LOD and the second part estimating the mean of the longitudinal response conditional on LOD being exceeded. The dependency between the two-parts of the longitudinal hurdle model was accounted for through the correlated random effects, which follow a multivariate normal distribution.

Graham et al. [66] considered using a longitudinal Tobit model (non-varying coefficient) for modelling the longitudinal outcome when some measurements achieved the highest limit. Lu et al. [27], Lu [38] and Lu et al. [80] proposed mixed-effects varying-coefficient models, and spline approaches are used to model the random effects and the population-level effects. They modelled the changing relationship between HIV viral load and CD4 cell counts in AIDS studies during the course of treatment.

##### Modelling multiple change points

In some clinical studies, multiple change points of each individual trajectory could occur due to variety of reasons. For example, in a study of HIV, the individual trajectories often have multiple points of rapid change due to the treatment effect [40]. Hennessey et al. [13], and Yu and Ghosh [55] considered a random change point model that accounts for trend in different individual trajectories, whereas Ghosh et al. [40] proposed a multiple-change point model which allows several up-and-down phases in the longitudinal marker trajectory.

##### Modelling longitudinal data with hierarchical structure

Generally, the longitudinal data have two level hierarchical structure where the individual is the only clustering factor. However, Brilleman et al. [81] employed GLMM to longitudinal data having a hierarchical structure with clustering factor beyond the individuals. They have modelled data from lung cancer where the interest was to study the relationship between tumour burden and risk of death or progression of disease. The longitudinal outcome was clustered within a specific tumour lesion for a given patient at a number of time points.

#### Count outcome

In modelling longitudinal count data with exceeded number of zeros, Hatfield et al. [53] proposed a two-part zero-augmented beta model (ZAB). Zhu et al. [31] proposed two zero-inflated count models, namely zero-inflated Poisson (ZIP) and zero-inflated negative binomial (ZINB). The latter differed from the former in having an additional parameter which captures the variability due to over-dispersion.

#### Random effect distribution

The longitudinal random effects are generally specified as following a normal distribution, see Table 1 and Table 2. In HIV studies however, outliers may occur among repeated measurements within an individual and some individuals may show very different behaviour from the rest. Distinguishing between these types might not be easy in practice [23]. Thus some articles employed a normal/independent (N/I) distribution as it has been shown through simulated studies that it is more robust for outliers than the conventional normal distribution [23, 35]. Baghfalaki et al. [48] has proposed a finite mixture of normal distributions to capture the unobserved heterogeneity of the random effects [48]. In a HIV study, using a simple exploratory diagnostic tool proposed by Verbeke and Molenberghs [90], they found that a finite mixture of normal distributions could improve the estimation when compared to the standard normal distribution. In some cases, a Dirichlet process prior is assigned to the random effects to allow for flexibility and avoid misspecification of the random effects distribution [40]. Martins et al. [82] assumed normally distributed random effects within different geographical regions to model the longitudinal outcome in a HIV study.

### Multivariate longitudinal outcomes (***K >*1**)

Multiple longitudinal outcomes were considered in 19 articles. Eight presented methods where all longitudinal outcomes were the same type of data (continuous outcomes [21, 28, 63], count outcomes [50], or ordinal outcomes [26]) whilst other 11 articles presented methods when the longitudinal outcomes were a mix of data types (e.g. continuous, ordinal and binary longitudinal outcomes [30]).

#### Continuous outcomes

For continuous data, generally multivariate mixed effect models were used [21, 28, 63, 83] and were described as in (1) for each *k*. The model accounted for two sources of dependency; within-individual repeated measurements over time for a given longitudinal outcome and between different longitudinal outcomes for the same individual.

Rue et al. [49] modelled two continuous longitudinal outcomes; an LME model was employed for first outcome and a mixed-effects beta regression model for the second outcome (a proportion). In the former, linear combinations of a cubic splines basis functions were considered to model the trajectory function to account for multimodal trends. The correlation between the two longitudinal outcomes was accounted through jointly modelling the individual specific random effect in each longitudinal outcome [49].

Tang and Tang [32] considered a partially LME model with spline terms to account for the complex functional structure between measurement times within and between outcomes. They used a P-spline approximation. Chen et al. [20] considered a GLM model and the trajectory function was allowed to take a linear or quadratic form based on the trend of mean response. Liu and Li [84] considered a zero-one inflated beta (ZOIB) regression model to account for [0, 1] interval data. Usually, beta distribution is known for offering a wide range of distributional shapes in the open support interval (0, 1).

#### Rate outcomes

A Zero-Augmented Beta (ZAB) model was considered for rate outcomes. The data are on a bounded measurement scale of [0, 1] interval, and a high number of zero longitudinal observations is included [50]. The model was expressed as,

6 $$ {Y}_{ik}\left({t}_{ij}\right)\sim ZAB\left({\omega}_i\left({t}_{ij}\right),{\mu}_i\left({t}_{ij}\right),\phi \right) $$

where *ω*_*i*_(*t*_*ij*_), *μ*_*i*_(*t*_*ij*_), and *ϕ* are the probability, mean and dispersion of *Y*_*ik*_(*t*_*ij*_) ∈ (0, 1) for the *k* th longitudinal outcome, respectively. A logistic model was assumed for *ω*_*i*_, and a beta regression model was assumed for *μ*_*i*_, and logit link function was used to estimate the corresponding parameters.

#### Count outcomes

In terms of multiple ordinal outcomes, Armero et al. [26] employed a proportional-odds cumulative logit model based on a continuous latent variable and was written as

7 $$ {Y}_{ik}\left({t}_{ij}\right)={D}_K\kern0.5em \Longleftrightarrow {Y}_{ik}^{\ast}\left({t}_{ij}\right)\in \left({\theta}_{k-1},{\theta}_k\right] $$

where ${Y}_{ik}^{\ast}\left({t}_{ij}\right)$ denotes the continuous latent variable and *D*_*K*_ represent a an ordinal category. A logistic distribution was proposed for ${Y}_{ik}^{\ast}\left({t}_{ij}\right)$ and used a mixed effect model for the individual-specific time trajectories of the latent variable. The translation of the ordinal variable through the latent variable offered flexibility in relation to the computational implementation of the model.

### Mixing type of longitudinal outcomes

A multivariate GLM model is often utilised when having a mixture of longitudinal outcomes with a link function for each outcome dependent on the type of data [14, 34, 36, 57]. Rizopoulos and Ghosh [57] proposed modelling the linear predicator using spline-based approach to allow flexibility in the individual-specific evolution for each outcome. The choice of link function used in the model depends on the distribution of the outcome. For example, an identity link function is utilised for a continuous outcome which follows a normal distribution, a logit link function is used if the outcome is binary and a log link function is applied when the outcome is a count.

Wang and Luo [85] employed a multidimensional latent trait linear mixed (MLTLM) model to allow for multiple latent variables and within-outcome multidimensionality in multiple longitudinal outcomes. However, Chen and Luo [86] considered a multilevel item response theory (MLIRT) model to account for skewness and outliers in the continuous outcomes. They assumed a heavy-tailed skew-normal/independent (SN/I) distribution. He and Luo [30] modelled a mixture of continuous, ordinal and binary longitudinal outcomes using MLIRT model. Wang et al. [87] proposed a semiparametric multilevel latent trait model (MLLTM) to simultaneously model continuous, binary and ordinal outcomes. A smooth time function based on truncated power series spline was included in the model to allow for additional flexibility.

However, Andrinopoulou et al. [45], Andrinopoulou et al. [29], and Baghfalaki et al. [41] proposed using a different model for each outcome and then link the models through a correlation structure, for example through random effects or measurement error. Andrinopoulou et al. [45] considered using a GLM model for the continuous data, whereas a continuation ratio mixed-effects model was proposed for the ordinal outcomes. Andrinopoulou et al. [29] proposed a mixed-effect model with B-spines to capture the complex trend in the continuous outcome, and a continuation ratio (CR) mixed-effects model was used when the individuals are likely to shift from one category to another. Baghfalaki et al. [41] considered using a LME model for continuous data whereas a continuous latent variable model was proposed for the ordinal longitudinal outcomes.

#### Random effect distribution

The random effects are generally assumed to follow a multivariate normal distribution [14, 20, 21, 28–30, 36, 41, 45, 49, 50, 63, 83, 84, 86, 87]. However, in the case of unspecified distribution of the random effects, a normal prior [26] or Dirichlet process prior [32, 34, 57] is assumed. Tang and Tang [32] conducted a simulation study to show the effect of the misspecification of the random effect distribution on the estimation, and found that assuming Dirichlet process prior reduces the bias and it is flexible enough to capture the general shapes of different distributions of the random effect.

#### Correlation structure

The correlation between the multiple longitudinal outcomes was captured through the individual-specific random effects for each outcome in a multivariate distribution [29, 41, 45, 86]. However, the correlation between within an individual *i* for measurements of multiple longitudinal outcomes measured at the same time was captured through the error term *ε*_*ij*._~*N*_*k*_(0, Σ), and *b*_*ik*_~*N*(0, Ψ_*k*_) where the covariance matrix Σ captures the association between longitudinal measurements recorded at the same time and the term Ψ_*k*_ is a covariance matrix that describes the association between the random effects for the k-th outcome [63, 83]. The joint model suggested by Chi and Ibrahim [63] has separately accounted for dependence among repeated measurements for a given outcome and correlation between multiple longitudinal outcomes.

#### Time-to-event data sub-model

Let *T*_*i*_ indicates the observed failure time for an individual *i* where *T*_*i*_ = $\mathit{\min}\left({T}_i^{\ast },{C}_i\right)$ and where ${T}_i^{\ast }$ denotes the true event time and *C*_*i*_ represents the censoring time. Let ${\delta}_i=I\left(\ {T}_i^{\ast}\le {C}_i\right)$ is an indicator taking value 0 if the response is censored and 1 if the event of interest is observed. A common choice for modelling the time-to-event (or event-time) data in the joint model is through the Cox proportional hazard model

8 $$ {\lambda}_i(t)={\lambda}_0(t)\exp \left\{{X}_i(t)\beta +{W}_i(t)\right\} $$

where *λ*_0_(*t*) is the baseline hazard function, *W*_*i*_(*t*) is a latent process, and *X*_*i*_(*t*) are covariates with the corresponding coefficients *β*. Several models are proposed for modelling the event-time outcomes; 42(66.7%) articles considered a single event-time outcome while 21(33.3%) articles proposed joint models for multiple event-time outcomes.

Although three types of censored event-times can occur, namely right, left and interval, in the review we have not found articles dealing with left censoring [91]. The right censored event-times occurs when the study period of the observation ends before the individuals experience the event. For example, if the event of interest is admission to the hospital, and by the end of the study, some individuals have not yet experienced this event. 27(42.8%) articles were based on right censoring. Left-censoring occurs when the event time is not observed but it is known to have happened before a certain time. When individuals experienced the event of interest within a known time period (e.g., between follow up appointments), they are interval-censored. Seven articles were based on interval-censored event-times [44, 51, 59, 60, 69, 70, 92]. For example, if an individual experienced a heart attack between the last two follow up appointments, it is known that the event of interest has happened, but it is not known exactly when it is happened. Su and Hogan [64] developed a joint model for doubly interval-censored event-times, accounting for the time between initiation of highly active antiretroviral therapy (HAART) and viral suppression (related to longitudinal CD4 count). The doubly interval-censoring occurred when both the time origin (HAART initiation) and failure time (viral suppression) were interval censored.

### Single event outcome

#### Semiparametric model

The majority of articles (42, 66.7%) were based on a single event outcome. Generally, the Cox proportional hazards model was employed [15, 17, 30, 31, 35, 39, 40, 46, 52, 76, 77, 79, 81, 83–87]. Yang et al. [24], and Waldmann and Taylor-Robinson [60] used a proportional hazards model which accounted for the quantile term defined in equation (3) in the model. This model enables to study the association of each longitudinal quantiles with the event-time outcome separately.

#### Full parametric model

12Fully parametric models were proposed to model the event-time data using Weibull distribution [19, 23, 33, 36, 43, 48, 50, 53]. Graham et al. [66] assumed that the event-time outcome follows a normal distribution with the mean depending on covariates and random effects in a study of dementia, where the time to death was the outcome of interest.

#### Accelerated failure time model (AFTM)

Modelling the event-time data is considered by adapting AFTM, which involved covariates that might affect the expected event time. Dagne [44] and Huang et al. [51] considered random effect AFTM for modelling the interval-censored event-time outcome and specified the error term to follow a normal distribution. Baghfalaki et al. [41] used log-normal distribution and Weibull distribution, whereas Huang and Chen [59], and Huang et al. [78] proposed a nonparametric Dirichlet process (DP) prior as a distribution for the error term in AFTM.

#### Relative risk model

Relative risk models have been used to model event-time outcomes by Andrinopoulou et al. [62], Rizopoulos and Ghosh [57], Armero et al. [26] and Martins et al. [82]. Andrinopoulou et al. [62] used a B-spline approach for time-varying coefficients that links the longitudinal and event-time outcomes. Armero et al. [26] proposed a left-truncated relative risk model to account for delays in the entry times of event-times. Rizopoulos and Ghosh [57] employed a relative risk model to examine the risk of graft failure in study of chronic kidney disease. Martins et al. [82] proposed a relative risk model to deal with spatial survival effects accounting for the unobserved heterogeneity among individuals living in the same geographical region.

#### Cure fraction in the time-to-event model

Modelling event-time data with cured individuals in a study population cannot be accomplished using model such as a proportional hazards model. Therefore, cure rate model is used, which is a special case of survival models where a portion of individuals in the population never experience the event of interest [20, 21, 47, 65]. Chi and Ibrahim [21] extended the model which allowed to accommodate for both zero and nonzero cure fraction with a proportional hazards structure.

### Multiple event-time outcomes

Multiple events occur when there are more than one event-time outcome of interest, for example, competing risks or recurrent events. Six (9.5%) methodological articles considered joint models for multiple event outcomes. The Cox proportional hazards model was commonly used to model the multiple event-times [14, 28, 32, 34, 38].

Chi and Ibrahim [63] proposed a novel bivariate survival model that has a proportional hazards structure for the population hazard when the baseline covariates are entered biologically through the mean function of the Poisson process. In many applications, such as cancer with multiple failure times (i.e. death and relapse), there is an interest to examine the joint or marginal survival distribution. Also, the marginal survival distribution accommodated both zero and nonzero cure fractions for the event-time, and in the joint survival distribution, an individual-specific frailty term is incorporated to capture the correlation between the two event-time outcomes.

Competing risk event-times are present when individuals are at risk of experience more than one mutually exclusive events, such as death from different causes. Fifteen (23.8%) methodological articles developed joint models for competing risk outcomes. Modelling of the competing risks is mostly carried out by a caused specific proportional hazards model [16, 18, 22, 27, 29, 37, 45, 49, 56, 61, 75]. Hennessey et al. [13] proposed modelling of time-to-dropout for various reasons by considering lognormal survival regression model to account for the dropout occurring in the early phase of the study. To account for substantial measurement error in the covariates when modelling competing risks, Lu [25] and Lu et al. [80] considered a cause-specific varying-coefficient proportional hazard model. Yu and Ghosh [55] considered a mixture of Weibull models to account for competing risks of dementia and dementia-free death.

### Baseline hazard function

The baseline hazard function was usually defined by a piecewise constant [14, 15, 17, 24, 29–32, 34, 38–40, 52, 60, 85, 86], while others used a step function [16, 18, 35, 75, 76]. Also, B-splines approach is utilised in many articles for specifying the baseline hazed function [22, 27, 37, 45, 46, 49, 56, 61, 81]. The baseline hazard function was also modelled parametrically by Weibull [19, 23, 43, 50, 53, 82] or by using an exponential distribution [36]. Andrinopoulou et al. [62] approximated the baseline hazard function using P-splines [93] and Wang et al. [87] and Tang et al. [28] adapted penalized splines for the baseline hazard.

### Association structure

In the joint model, the longitudinal and event-time outcomes are linked by an association structure. The association structure represents the effect of longitudinal outcome(s) on the risk of event(s). The choice of association structure should be made based on the clinical background of the study. However, this information may not always be accessible, and therefore, Rizopoulos and Ghosh [57] evaluated several association structures, and identified reasons for using the different association structures. More details for choice of association structures can also be found in the article by Hickey et al. [1].

A linear combination of individual-specific random effects were used to define the association structure in 39(52%) articles. The current value association structure is commonly used in the joint model with 27(36%) articles using it. Many other structures have been proposed to link the two sub-models including the correlated random effects (3, 4%), time-dependent slope (3, 4%), random effects with fixed effect (2, 2.7%) and cumulative effect (1, 1.3%). Table 3 summarises the proposed association structures.

**Table 3:** Association structures for joint model

Parameterisation	Latent association		Number of articles (%)^a^	Reference
Random effect	Univariate	*W*_*i*_(*t*) = *αb*_*i*_	31(41%)	[13, 15, 17, 19, 22, 23, 25, 27, 31, 33, 35, 37, 38, 44, 48, 51, 53, 54, 56, 59–61, 66, 69, 70, 75, 77–80, 82]
	Multivariate	${W}_i(t)={\sum}_{k=1}^K{\alpha}_k{b}_{ik}$	8(10.7%)	[20, 30, 36, 41, 50, 82, 85–87]
Current Value parameterisation	Univariate	*W*_*i*_(*t*) = *αm*_*i*_(*t*)	14(18.7%)	[13, 24, 39, 40, 43, 46, 47, 52, 65, 67, 71–73, 76]
	Multivariate	${W}_i(t)={\sum}_{k=1}^K{\alpha}_k{m}_{ik}(t)$	13(17.3%)	[14, 21, 26, 28, 32, 34, 45, 49, 57, 63, 83, 84, 87]
Correlated random effect	Univariate	*W*_*i*_(*t*) = *φ* with ϐ_*i*_ = {*b*_*i*_, *φ*_*i*_}~*H*	2(2.7%)	[16, 18]
	Multivariate	*W*_*i*_(*t*) = *φ* with ${\mathrm{\ss}}_i=\left\{{b}_i,{\varphi}_i\right\}\sim {H}_{\alpha_k}$	1(1.3%)	[57]
Random effect with fixed effect	Multivariate	${W}_i(t)={\sum}_{k=1}^K{\alpha}_k\left({\beta}_k+{b}_{ik}\right)$	2(2.7%)	[29, 57]
Time-dependent slope	Univariate	${W}_i(t)={\alpha}^{(1)}{m}_i(t)+{\alpha}^{(2)}\ \frac{d\ }{dt}{m}_i(t)$	1(1.3%)	[87]
	Multivariate	${W}_i(t)={\sum}_{k=1}^K\left\{{\alpha_k}^{(1)}\ {m}_{ik}(t)+{\alpha_k}^{(2)}\ \frac{d\ }{dt}{m}_{ik}(t)\right\}$	2(2.7%)	[45, 81]
Cumulative effect	Univariate	${W}_i(t)=\alpha {\int}_0^t{m}_i(s) ds$	1(1.3%)	[45]

Abbreviation: ^a^The number of article that used this association among all other articles with its percentage

Notation: *m*_*ik*_(*t*) denotes the true underlying value of the longitudinal outcome for individual *i* and outcome *k*; *α*_*k*_ represents the association parameter for the *k*-th outcome; *α*_*k*_^(1)^ and *α*_*k*_^(2)^ denote the association parameters for the current value and the derivative from the mean trajectory function for the *k*-th longitudinal outcome respectively; *b*_*ik*_ denotes the random effect for individual *i* and outcome *k*; *φ* represents a random effect and *H* denotes joint distribution for the random effects; *β*_*k*_ denotes the coefficient parameters

Brilleman et al. [81] assumed patient–level summary measures were associated with the hazard of the event in hierarchical structure data. Examples of patient–level summary measures are average, maximum, or minimum of functions of the longitudinal sub-model parameters (i.e. the lower level cluster-specific linear predictor or rate of change in the marker at time *t*).

Several authors proposed using a variety of parameterisation, and then examined the influence of each parameterisation on the model estimation [13, 45, 49, 57, 87], see Table 3. These parametrisations were compared using an information criterion such as DIC, to find the best association structure for making inference and prediction. However, Andrinopoulou et al. [62] assumed that the effect of the longitudinal outcome might have a time-varying effect on the time-to-event outcome, and a B-spline approach was employed to model the association parameter. They have considered current value, time-dependent slope, and cumulative effect association structures with time-varying coefficient defined respectively as *W*_*i*_(*t*) = υ(*t*)*m*_*i*_(*t*), ${W}_i(t)={\upupsilon}_1(t){m}_i(t)+{\upupsilon}_2(t)\ \frac{d\ }{dt}{m}_i(t)$, and ${W}_i(t)=\upupsilon (t){\int}_0^t{m}_i(s) ds$ where *m*_*i*_(.) denotes the true underlying value of the longitudinal outcome, $\upupsilon (t)=\sum \limits_{l=1}^L{\alpha}_l{B}_l(t)$, *α*_*l*_ is a set of parameters that capture the strength of the association between the two outcomes, and *B*_*l*_(*t*) represents the *l*-th basis function of a B-spline.

### Alternative approaches to joint model

Several alternative approaches to shared parameter joint models are identified in the review.

### Latent class joint model

Joint latent class models assume that the population in the study is heterogeneous and is constructed of a number of latent subgroups that are homogeneous [94]. Hence, each class shared the same mean trajectory function and hazard function. For class *p*, the longitudinal outcome is defined by class-specific mixed-effect sub-model and the event-time outcome is defined by class-specific proportional hazards sub-model

9 $$ {Y}_i\left({t}_{ij}|{c}_i=p\right)={X}_i\left({t}_{ij}\right){\beta}_p+{Z}_i\left({t}_{ij}\right){b}_{ip}+{\varepsilon}_{ij} $$

10 $$ {\lambda}_i\left(t|{c}_i=p\right)={\lambda}_{0\mathrm{p}}(t)\exp \left({X}_i(t){\beta}_p+{W}_{ip}(t)\right) $$

where *c*_*i*_ is latent class indicator for the *i* th individual, and other parameters are defined similarly as in general sub-models. To identify the number of classes, the Bayesian Information Criterion (BIC) is adapted [94].

The above sub-models were considered by Andrinopoulou et al. [68] when both the longitudinal and event time outcomes are single. When the longitudinal outcome is measured with limit of detection (LOD), Huang et al. [54] proposed a class-specific nonlinear mixed-effect Tobit model for the longitudinal outcome, and an accelerated failure-time model was used for the event-time outcome. Dagne [69] considered a two-part Tobit model for longitudinal outcome which accounted for left-censored outcome and heterogeneity among individuals. They have also used an accelerated failure model for the class specific event-time outcome. Also, to adjust for the skewness in the data, Dagne [69] assumed a multivariate skew-t (ST) distribution for the random error. Garre et al. [67] modelled the longitudinal outcome using an intercept-only random-effects model and a segmented random change-point model. To model a non-linear pattern in longitudinal outcome, Chen and Huang [70] considered a mixture of semiparametric mixed-effects models under multivariate ST distribution. Entink et al. [58] proposed a mixture multilevel item response model. In modelling nonlinear heterogeneous multivariate longitudinal data, Huang et al. [42] considered using a finite mixture of nonlinear mixed-effects models for modelling the latent class in the longitudinal trajectories. They have proposed modelling of multiple event outcomes using proportional hazards where the hazard function for each latent class is defined as step function [42].

### Functional joint model

The functional joint model approach involves modelling the longitudinal outcome, event-time outcome and exposure variables that include both scalar predictors and functional predictors. The functional predictors consist of a sample of functions that have information about curves, surfaces, or other geometric features that are varying over time [73]. These types of function are defined on a one-dimensional time domain, e.g. growth curve data and heart rate monitor data. The functional longitudinal model can be expressed as

11 $$ {Y}_i\left({t}_{ij}\right)={X}_i\left({t}_{ij}\right)\beta +{\int}_S^g{g}_i^{(x)}(s){B}^{(x)}(s) ds+{Z}_i\left({t}_{ij}\right){b}_i+{\varepsilon}_{ij} $$

where ${g}_i^{(x)}(s)$ is a time-invariant function predictor defined over a one-dimensional domain *S*, and the coefficient function *B*^(*x*)^(*s*) denotes the pointwise association between the functional predictor and the longitudinal outcome. This approach was proposed to model the growing volume of functional data, collected in higher dimensional domains in both longitudinal and event-times outcomes [73]. The function event-time outcome model can be defined as

12 $$ {\lambda}_i(t)={\lambda}_0(t)\exp \left({X}_i(t)\beta +{\int}_S^g{g}_i^{(x)}(s){B}^{(x)}(s) ds+{W}_i(t)\right) $$

where the term ${\int}_S^g{g}_i^{(x)}(s){B}^{(x)}(s)$ represents the functional predictor. The inclusion of this term aims to show the influence of the functional predictor toward the event hazard.

To model longitudinal functional, longitudinal scalar and event-time outcomes simultaneously, Li and Luo [74] proposed a multivariate functional joint model. Modelling of the functional longitudinal data was carried out by adapting a functional mixed effect model, and a functional principle component analysis (FPCA) approach was used to expand the random intercept function. FPCA is a dimension reduction tool.

### Additive joint model

Additive joint models involve a highly flexible specification of the association between the longitudinal outcome and an event-time outcome process. Köhler et al. [71] proposed an additive joint model which is allowed for complex nonlinear association structures between the longitudinal and the event-time outcome processes. Kohler et al. [72] considered an additive joint model using penalized splines for longitudinal trajectory with a potentially nonlinear time varying association structure.

### Bayesian estimation

The Bayesian approach works by estimating the joint posterior distribution of the model, which is a product of the joint likelihood of the longitudinal and event-time outcome data and the joint prior distribution. The latter includes prior information that can be assigned for the unknown parameters in the joint model [24]. The joint posterior distribution can be written as

$$ p\left(\theta, {b}_i|{Y}_i,{T}_i\right)\propto L\left({Y}_i,{T}_i|\theta \right)p\left(\theta \right) $$

where the term *L*(*Y*_*i*_, *T*_*i*_| *θ*) is the joint likelihood of the longitudinal and event-time outcome data and *p*(*θ*) denotes the prior information of the unknown parameters *θ* in the joint model. The term *θ* represents all parameters to be estimated from the model, while *T*_*i*_ denotes the event-time, *Y*_*i*_ is the longitudinal data and *b*_*i*_ are the random effects as defined in sub-models.

The Bayesian sampling algorithms used to estimate *θ* are summarised in Table 4. The sampling algorithms work by drawing samples from the joint posterior distribution [24]. A total of 66(91.6%) articles used Markov Chain Monte Carlo (MCMC), of which 37(54.4%) specified the sampling algorithm used: Gibbs sampling 9(12.5%), Gibbs sampler and Metropolis-Hastings (MH) algorithms 24(33.3%), Gibbs sampling with adaptive rejection algorithm and MH sampling 3(4.2%), and block Gibbs sampling and MH algorithm 2(2.8%). 28(38.8%) articles did not specify the algorithm used. Köhler et al. [71] used Newton-Raphson procedure and a derivative-based Markov chain Monte Carlo (MCMC) algorithm in order to estimate the mode and the mean of the posterior distribution. Li and Luo [73] and Li and Luo [74] considered No-U-Turn sampler instead of Gibbs sampler since it was faster to converge. Brilleman et al. [81] employed Hamiltonian Monte Carlo (HMC) instead of an MCMC method for its ability to explore the parameter space of the posterior distribution more efficiently. A combination of HMC and No-U-Turn sampler was adapted by Wang et al. [87] since it resulted in faster convergence compared to HMC alone. Tang et al. [28] used the Bayesian Lasso to simultaneously estimate the parameters and select the important covariates in model.

**Table 4:** Bayesian sampling algorithms

Sampling algorithm	Number of articles (%)	Reference
Markov Chain Monte Carlo (MCMC)	28(38.8%)	[13, 17, 20, 24, 26, 30, 31, 33, 38, 39, 43, 46, 49, 50, 53, 57, 58, 60, 62, 67, 68, 72, 75, 76, 82, 84–86]
Gibbs sampler and Metropolis Hastings (MH)	24(33.3%)	[14, 15, 22, 23, 25, 27, 29, 35, 37, 41, 42, 44, 48, 51, 54, 56, 59, 61, 69, 70, 77–80]
Gibbs sampling	9(12.5%)	[19, 36, 40, 45, 47, 52, 65, 66, 83]
Gibbs sampling with adaptive rejection and MH	3(4.2%)	[16, 18, 63]
Block Gibbs sampling and MH	2(2.8%)	[32, 34]
Bayesian Lasso	1(1.4%)	[28]
Newton-Raphson procedure and a derivative-based MCMC	1(1.4%)	[71]
No-U-Turn sampler	2(2.8%)	[73, 74]
Hamiltonian Monte Carlo (HMC)	1(1.4%)	[81]
HMC and No-U-Turn sampler	1(1.4%)	[87]

Assessing the convergence of MCMC is essential when employing the Bayesian estimation. The diagnostic tools have been designed to evaluate how long the chain takes to produce observations from the stationary distribution of the Markov chain [95]. The trace plots, Gelman–Rubin statistics, autocorrelations plot and the potential scale reduction factor (PSRF) were used in reviewed article.

### Prior and sensitivity analysis

One of the advantages of Bayesian estimation is the ability of incorporating information from previous studies through prior distributions of parameters. The incorporated prior could be informative or non-informative. The latter is employed in the absence of prior information or when there is no need to influence the model fit with any prior information about the parameters, and therefore this type of prior has a minimal influence on the estimation. The former is assigned when some information is available from previous research which probably have an impact on the posterior distribution. It is necessary to check the influence of the assigned prior on the posterior estimation by performing a sensitivity analysis [38, 56].

Generally, a weakly or non-informative normal prior is assumed for the fixed effect parameters in the longitudinal model. Brown and Ibrahim [52] assumed a Dirichlet process prior for the longitudinal model parameters to allow for more flexible modelling framework since not all of the longitudinal parameters come from the same distribution and these parameters might not remain constant over time. Chen et al. [39] assigned a uniform improper priors.

The unknown fixed effect parameters in event-time sub-model are generally assumed to follow a normal weakly or non-informative prior distribution Choi et al. [36] specified a multivariate normal distribution for the event-time fixed effect coefficients whereas Brilleman et al. [43] assumed Cauchy priors.

Generally, the association parameter is assigned to follow a normal weakly or non-informative prior distribution. Das et al. [76] assumed a uniform prior for the association parameter.

In Bayesian estimation, as prior information about the parameters is included in the model, it is important to check the sensitivity of the incorporated prior on the estimation. In many articles, influence of the assigned priors on the posterior estimation was carried out by trying different hyper-priors [15, 20, 22, 24–27, 31, 32, 37, 38, 41, 56, 61, 76, 78–80, 82]. Zhu et al. [14] developed a Bayesian influence approach aimed to assess the sensitivity of inference to different unverifiable assumptions under the framework of Bayesian analysis of joint models and to detect influential observations or outliers.

### Dynamic prediction

Using the available information to provide risk assessment of a disease or predict a future longitudinal measurement is valuable in clinical studies. Dynamic prediction is based on updating the prediction from the joint model as new survival or longitudinal information is recorded [96].

Armero et al. [26], Li and Luo [73] and Wang et al. [87] proposed a dynamic prediction for future longitudinal measurements and estimated the survival function of patients at future time point *u*. Choi et al. [36] generated dynamic predictions from the probabilities of events that happen within a fixed window of time, while Yang et al. [24] considered predicting the survival probability of new patients up to time *u*. Andrinopoulou et al. [45] considered predicting the cumulative incidence probabilities for a new patient using multiple longitudinal measurements. Li and Luo [74] generated dynamic predictions of scaler and functional outcomes at future time point as well as the conditional probability of event-free at a future time *u*.

Andrinopoulou et al. [45] and Rizopoulos et al. [46] proposed using Bayesian model averaging (BMA) approach to combine predictions from different joint models based on different association structures to provide more efficient risk predictions. This approach accounted for model uncertainty and not all the individuals have the same prognostic model.

### Software

To implement the algorithms, a variety of software have been utilised, as shown in Table 5. A total of 21 articles (36.8%) fitted joint models through WinBUGS programme (MRC Biostatistics Unit, Cambridge, UK). Eleven articles provided the code to fit the model: four were available on request from the authors [15, 51, 77, 78], six were available in the appendix or supplement materials [13, 17, 37, 48, 57, 66] and one could be accessed online [33]. In two articles, both OpenBUGS and BUGS languages were used to develop codes, but only one provided the code in the appendix [31].

**Table 5:** Software used with Bayesian joint models

Software	Number of articles (%)	Reference
WinBUGS	21(35.6%)	[13, 15, 17, 22, 23, 27, 33, 37, 41, 42, 44, 48, 51, 56, 57, 61, 66, 67, 77, 78, 80]
OpenBUGS	2(3.4%)	[31, 82]
BUGS language	1(1.7%)	[30]
R	5(8.5%)	[39, 60, 68, 71, 72]
R (interface to WinBUGS)	10(16.9%)	[19, 35, 42, 50, 53, 54, 59, 69, 70, 79]
R (interface to OpenBUGS)	1(1.7%)	[36]
R (interface to JAGS)	1(1.7%)	[84]
R and JAGS	2(3.4%)	[45, 46]
R and Matlab	1(1.7%)	[34]
R and WinBUGS	1(1.7%)	[29]
JAGS	3(5%)	[24, 26, 49]
Stan	6(10%)	[43, 73, 74, 81, 85, 87]
Matlab	1(1.7%)	[28]
C language	3(5.1%)	[25, 40, 52]
Fortran	1(1.7%)	[83]

The R software [97] was employed in 21 articles (35.6%), 10 had access to WINBUGS (using R2WinBUGS package) [19, 35, 42, 50, 53, 54, 59, 69, 70, 79], one got access to OpenBUGS (through rbugs package) [36] and one used an R interface to JAGS through the R package rjags [84]. Several articles used existing packages (such as JMbayes and bamlss) [71, 72] to fit the model, whereas others developed their own R software, which were available in the appendix [42, 54, 70] or could be requested from the corresponding author [35, 36, 59, 60, 79].

Other software used in review articles included JAGS [98], Fortran [83], Stan and C language, with codes available upon request from author [40, 52] or in the supplementary material [24, 43, 73, 74, 81, 85–87].

Andrinopoulou et al. [29] implemented the algorithm using two software programmes, WinBUGS and R. Rizopoulos et al. [46] and Andrinopoulou et al. [45] used R and JAGS to implement the algorithms, where Tang et al. [34] used R and Matlab (The MathWorks Inc., Natick, MA) and the codes can be requested from the author.

### Application

A total of 14 application articles were found in the review where Bayesian joint modelling approaches have been considered to tackle issues in data, or answer questions regarding the relationship between the longitudinal biomarkers and event-time outcomes [2–6, 92, 99–106]. They were applied in a wide range of disease areas; cancer [5, 6, 99], HIV/AIDS studies [2–4], cystic fibrosis [100], renal disease [101], diabetes [102], cognitive functioning [104], Huntington’s disease [105], and eye disease [106], and also other research areas such as health insurance [103] and daily living [92]. For example, Serrat et al. [5] used the joint model to show the association between the prostate-specific antigen and the risk of prostate cancer. Khoshhali et al. [101] determined the relationship between serum albumin levels and peritoneal dialysis technique failure in patients according to diabetic status. Guure et al. [104] wanted to examine and assess the association between Mental State Examination and the risk of dying due to cognitive impairment. Long and Mills [105] aimed to estimate a multivariate joint model using data from four longitudinal observational studies and compute individual-specific predictions for characterising progression of Huntington’s disease. There were two longitudinal outcomes: total motor score and the symbol digit modalities test, and the event-time was time to motor diagnosis.

## Discussion and conclusion

A large number of approaches have been proposed and employed to model longitudinal and event-time outcomes jointly. The first article of this review was published in September 2003. Mixed effect models were the most common modelling approach for longitudinal data, while the Cox proportional hazards model was commonly used to represent the event-time outcome. A wide range of models including cure rate model, Bent-cable mixed-effects model and proportional-odds cumulative logit model were employed in the literature to handle different type of longitudinal and event-time outcome data. For example, several articles proposed modelling event-time data with cured individuals using a cure rate model instead of a proportional hazards model. The cure rate models can account for a special case of survival models where a portion of individuals in the population never experience the event of interest.

In general in Bayesian joint models, the prior is defined for the unknown fixed effect parameter and the association parameter. The model incorporates prior information from previous studies to influence the posterior distribution. However, in some articles, a prior was also assumed for both fixed and random effect parameters in longitudinal trajectory, which can offer more flexibility in modelling the longitudinal trajectory and avoiding the uncertainty regarding the distributional assumptions. A Dirichlet process prior is the most popular, which can be used to create a family of distributions to provide more flexible priors than the standard normal distribution.

MCMC is generally adapted to estimate the parameters in Bayesian joint modelling approaches. A posterior mean is usually estimated using the MCMC, however, in a couple of articles, the mode was also estimated in addition to the mean of posterior distribution using Newton-Raphson procedure and a derivative-based MCMC algorithm. The speed of convergence was one of the factors considered in choosing an appropriate sampler in MCMC, for example, No-U-Turn sampler was chosen over the Gibbs sampler for the reason of fasting converge. The rationale behind the choice of MCMC sampler has not been justified in all the articles, therefore, development of appropriate reporting guidelines would be beneficial for the use of MCMC or its extensions, and the type of sampler to be used in different scenarios when modelling joint longitudinal and time-to-event data.

One of the recent advances of joint modelling is to generate dynamic predictions. This process updates the prediction from a joint model as new information becomes available. Several articles developed methods for providing dynamic predictions for survival probability and future longitudinal measurements. This characteristic is beneficial in medical research as it helps to provide a tailored disease progression for individuals, and therefore takes a relatively accurate decision to improve the health decision-making. For example, in the a heart valve replacement [45], such dynamic prediction tools can inform the physicians about future re-intervention to their patients. In this case, the available follow-up measurements of the current patients were utilized to produce updated predictions on survival and re-intervention in future. However, methods to validate these predictions are not yet fully developed. The validation measures are able to see how the model predicted the observed data (these are called calibration measure) and the ability of the model to discriminate between individuals who experienced the event and those did not (discrimination measure). Not all of the available validation methods, especially the calibration measures, can be utilised in real data. Only the discrimination measures such as the receiver operating characteristic (ROC) curves can be used for assessing predictive accuracy.

Many approaches assumed that the association parameter to be constant over time, however, in some study populations, the relation between the biomarkers trajectory and the risk of event might change over time. Only one article proposed a method along splines to account for such time dependant changes in association Andrinopoulou et al. [62].

A couple of articles discussed the effect of the chosen association structure on the analysis [13, 45, 49, 57, 87]. However, none proposed a method when the association between the two processes cannot be firmly specified from the data or clinical background.

In conclusion, we have reviewed a wide range of joint models in univariate and multivariate settings within Bayesian framework, summarising the model specifications, association structure, computing algorithm and dynamic prediction. We have also identified several future research directions for this area, including better methodologies for validation of dynamic prediction, modelling of time-varying association parameters, and techniques to account for unspecified association structure.

## Supplementary information

**Additional file 1.** This file includes the search strategies used in this review to search Medline, Scopus and Web of Science.

**Additional file 2.** This file includes a blank example of the data collection form used to record information from the studies identified by this review.

**Additional file 3.** This file includes the univariate and multivariate longitudinal models are illustrated in more detail for the studies identified by this review.

## Data Availability

Not applicable. No datasets were used in this study.

## Abbreviations

MCMC: Markov Chain Monte Carlo
MH: Metropolis-Hastings algorithms
HMC: Hamiltonian Monte Carlo
GLM: Generalized linear mixed model
LME: Linear mixed-effect
NLME: Nonlinear mixed-effects model
SNLME: Semiparametric nonlinear mixed-effects model
LQMM: Linear quantile mixed model
QR- based NLME: Quantile regression based nonlinear mixed-effects model
MLIRT: Multilevel item response theory model
MLLTM: Multilevel latent trait model
ZAB: Zero-augmented beta model
AFTM: Accelerated Failure Time Model
ZIP: Zero-inflated Poisson
ZINB: Zero-inflated negative binomial
IOU: Integrated Ornstein-Uhlenbeck stochastic process
CR: Continuation ratio
ALD: Asymmetric Laplace distribution
SN/I: Skew-normal/independent
SN: Skew-normal
ST: Skew-t
N/I: Normal/independent
DP: Dirichlet process
FPCA: Functional principle component analysis
LOD: Limit of detection
PSRF: Potential scale reduction factor
BMA: Bayesian model averaging
QTLs: Quantitative trait loci
