# Supplementary material for: Bayesian joint modelling of longitudinal and time to event data: a methodological review
Source: BMC Med Res Methodol. 2020 Apr 26;20:94. doi: 10.1186/s12874-020-00976-2 (PMC7183597; doi:10.1186/s12874-020-00976-2)
Supplement: Supplementary file 1 — Additional file 1. This file includes the search strategies used in this review to search Medline, Scopus and Web of Science. [file 12874_2020_976_MOESM1_ESM.docx]

**Additional file 1**

**Search strategy**

**Database - Scope**

( ABS ( joint  AND model )  AND  ABS ( Bayesian ) ) OR ( TITLE ( joint  AND model )  AND  TITLE ( bayesian ) ) OR ( ABS ( joint  AND models )  AND  ABS ( Bayesian ) ) OR ( TITLE ( joint  AND models )  AND  TITLE ( bayesian ) ) OR ( ABS ( joint  AND modelling )  AND  ABS ( Bayesian ) ) OR ( TITLE ( joint  AND modelling )  AND  TITLE ( bayesian ) ) OR ( ABS ( longitudinal  AND  survival )  AND  ABS ( Bayesian ) ) OR ( TITLE ( longitudinal  AND  survival )  AND  TITLE ( bayesian ) )

**Database - Web of Science**

Search Strategy:

1. **TITLE:** (Joint model) *AND* **TITLE:** (Bayesian)
2. **TITLE:** (Joint models) *AND* **TITLE:** (Bayesian)
3. **TITLE:** (Joint modelling) *AND* **TITLE:** (Bayesian)
4. **TITLE:** (longitudinal and survival) *AND* **TITLE:** (Bayesian)
5. 1 or 2 or 3 or 4

**Database - Ovid MEDLINE**

Search Strategy:

1. (Joint model and Bayesian).ab,ti.
2. (Joint models and Bayesian).ab,ti.
3. (Joint modelling and Bayesian).ab,ti.
4. (longitudinal and survival and Bayesian).ab,ti.
5. 1 or 2 or 3 or 4
