# Supplementary material for: Bayesian joint modelling of longitudinal and time to event data: a methodological review
Source: BMC Med Res Methodol. 2020 Apr 26;20:94. doi: 10.1186/s12874-020-00976-2 (PMC7183597; doi:10.1186/s12874-020-00976-2)
Supplement: Supplementary file 2 — Additional file 2. This file includes a blank example of the data collection form used to record information from the studies identified by this review. [file 12874_2020_976_MOESM2_ESM.docx]

**Additional file 2**

**Data extraction form**

| **General information** |  |
| --- | --- |
| Title |  |
| Authors |  |
| Journal |  |
| Year |  |
| Reference |  |
| Type of the article: Application or methodological article |  |
| Joint modelling method |  |
| Longitudinal model |  |
| Longitudinal variable(s) |  |
| Error distribution |  |
| Random effect distribution |  |
| Correlation structure |  |
| Time-to-event model |  |
| Time-to-event variable(s) |  |
| Censoring type |  |
| Association structured used |  |
| Estimation approach |  |
| Fixed effect Prior |  |
| Random effect Prior |  |
| Association parameter Prior |  |
| Software |  |
| Sensitivity analysis |  |
| Simulation study |  |
| Convergence diagnostic |  |
| Dynamic prediction |  |
| Clinical example |  |
