# Supplementary material for: Bayesian joint modelling of longitudinal and time to event data: a methodological review
Source: BMC Med Res Methodol. 2020 Apr 26;20:94. doi: 10.1186/s12874-020-00976-2 (PMC7183597; doi:10.1186/s12874-020-00976-2)
Supplement: Supplementary file 3 — Additional file 3. This file includes the univariate and multivariate longitudinal models are illustrated in more detail for the studies identified by this review. [file 12874_2020_976_MOESM3_ESM.docx]

**Additional file 3**

**Table 1A.Summary of longitudinal sub-models with single longitudinal outcome**

| **Outcome Type** | **Error distribution** | **Random effect distribution** | **Model** | **Reference** |
| --- | --- | --- | --- | --- |
| Continuous | Normal | Normal | LME | [19,33,46,75,76] |
| Continuous | Normal | Normal | Mixed effect model with IOU stochastic process | [47] |
| Continuous | Normal | Normal | GLM | [84] |
| Continuous | Normal | Normal | Random change point model | [13,55] |
| Continuous | Normal | Multivariate normal | LME | [16,17,62] |
| Continuous | Normal | Multivariate normal | Mixed effect model | [39] |
| Continuous | Normal | Multivariate normal | Longitudinal Tobit model | [66] |
| Continuous | Normal | Finite mixture of normal distributions | LME | [48] |
| Continuous | Normal | Dirichlet process prior | Semiparametric random-effects model | [52] |
| Continuous | Normal | Unspecified (a Dirichlet process prior) | Multiple-change point model | [40] |
| Continuous | Normal | Normal | Longitudinal model for the immune response | [65] |
| Continuous | N/I | N/I | LME | [23,35] |
| Continuous | SN | Normal | NLME | [51] |
| Continuous | t-distribution | Multivariate normal | LME | [18] |
| Continuous | ST | Normal | LME | [15] |
| Continuous | ST | Normal | Mixed-effects varying-coefficient model | [38] |
| Continuous | ST | Approximated by spline | Partially LME | [22,37,56,61] |
| Continuous | Multivariate ST | Normal | Mixed-effects varying-coefficient model | [27,83] |
| Continuous | Multivariate ST | Normal | Mixed-effect varying coefficient Tobit model | [25] |
| Continuous | Multivariate ST | Normal | NLME | [79] |
| Continuous | Multivariate ST | Multivariate normal | Bent-cable mixed-effects model | [44] |
| Continuous | Multivariate ST | Approximated by spline | SNLME | [80] |
| Continuous | ALD | Normal | QR-NLMET | [82] |
| Continuous | ALD | Multivariate normal | LQMM | [24] |
| Continuous | ALD | Multivariate normal | QR- NLME | [59] |
| Continuous | Not stated | Normal | Quantile-based mixed model | [60] |
| Continuous | Not stated | Multivariate normal | Hurdle two-part model | [43] |
| Count | NA | Multivariate normal | ZAB | [53] |
| Count | NA | Multivariate normal | Two zero-inflated count models | [31] |

**Table 2A. Summary of longitudinal sub-models with multivariate longitudinal outcome**

| **Outcome (s) Type** | **Error distribution** | **Random effect distribution** | **Model** | **Reference** |
| --- | --- | --- | --- | --- |
| Continuous | Normal | Normal | GLM | [20] |
| Continuous | Normal | Multivariate normal | LME and a mixed-effects beta regression model | [49] |
| Continuous | Multivariate normal | Multivariate normal | Multivariate mixed effect models | [21,63,77,86] |
| Continuous | Finite mixture of normal distributions | Multivariate normal | Multivariate mixed effect models | [28] |
| Continuous | Multivariate SN | Unspecified (a Dirichlet prior ) | Partially LME | [32] |
| Rate | NA | Normal | ZAB | [50] |
| Ordinal | NA | Normal | Proportional-odds cumulative logit model | [26] |
| Discrete(or/and continuous) | Multivariate normal (Continuous outcome) | Multivariate normal | Multivariate GLM | [14] |
| Discrete(or/and continuous) | Multivariate normal | Multivariate normal | ZOIB | [87] |
| Continuous, Ordinal and Discrete | Multivariate Normal (Continuous outcome) | Unspecified (a Dirichlet prior ) | Multivariate GLM | [34] |
| Continuous and binary | Normal (Continuous outcome) | Normal | Multivariate GLM | [36] |
| Continues and binary | Normal (Continuous outcome) | Unspecified (a Dirichlet prior ) | Multivariate GLM | [57] |
| Continuous and ordinal | Normal ( Continuous outcome) | Normal | GLM (Continuous outcome) and CR mixed-effects model (Ordinal outcome) | [45] |
| Continuous and ordinal | Normal (Continuous outcome ) | Normal | Mixed-effect model (Continuous outcome) and CR mixed-effects model (Ordinal outcome) | [29] |
| Continuous and ordinal | Normal (Continuous outcome ) | Multivariate Normal | LME (Continuous outcome) and continuous latent variable model (Ordinal outcome) | [41] |
| Continuous, ordinal and binary | Normal (Continuous outcome ) | Normal | MLIRT | [30] |
| Continuous, binary and ordinal | Normal (Continuous outcome) | Normal | MLTLM | [88] |
| Continuous, ordinal and binary | Normal (Continuous outcome) | Normal | MLLTM | [90] |
| Continuous, ordinal and binary | SN/I (Continuous outcome) | Normal | MLIRT | [89] |
